# Bovine Polymorphonuclear Neutrophils Cast Neutrophil Extracellular Traps against the Abortive Parasite *Neospora caninum*

**DOI:** 10.3389/fimmu.2017.00606

**Published:** 2017-05-29

**Authors:** Rodolfo Villagra-Blanco, Liliana M. R. Silva, Tamara Muñoz-Caro, Zhengtao Yang, Jianhua Li, Ulrich Gärtner, Anja Taubert, Xichen Zhang, Carlos Hermosilla

**Affiliations:** ^1^Institute of Parasitology, Justus Liebig University Giessen, Giessen, Germany; ^2^College of Veterinary Medicine, Jilin University, Changchun, China; ^3^Institute of Anatomy and Cell Biology, Justus Liebig University Giessen, Giessen, Germany

**Keywords:** neutrophil extracellular trap, nicotinamide adenine dinucleotide phosphate-oxidase, protein arginine deiminase 4, g protein-coupled receptor 2, *Neospora caninum*

## Abstract

*Neospora caninum* represents a relevant apicomplexan parasite causing severe reproductive disorders in cattle worldwide. Neutrophil extracellular trap (NET) generation was recently described as an efficient defense mechanism of polymorphonuclear neutrophils (PMN) acting against different parasites. *In vitro* interactions of bovine PMN with *N. caninum* were analyzed at different ratios and time spans. Extracellular DNA staining was used to illustrate the typical molecules of NETs [i.e., histones (H3), neutrophil elastase (NE), myeloperoxidase (MPO), pentraxin] *via* antibody-based immunofluorescence analyses. Functional inhibitor treatments were applied to reveal the role of several enzymes [NADPH oxidase (NOX), NE, MPO, PAD4], ATP-dependent P2Y2 receptor, store-operated Ca^++^entry (SOCE), CD11b receptor, ERK1/2- and p38 MAPK-mediated signaling pathway in tachyzoite-triggered NETosis. *N. caninum* tachyzoites triggered NETosis in a time- and dose-dependent manner. Scanning electron microscopy analyses revealed NET structures being released by bovine PMN and entrapping tachyzoites. *N. caninum*-induced NET formation was found not to be NOX-, NE-, MPO-, PAD4-, ERK1/2-, and p38 MAP kinase-dependent process since inhibition of these enzymes led to a slight decrease of NET formation. CD11b was also identified as a neutrophil receptor being involved in NETosis. Furthermore, *N. caninum*-triggered NETosis depends on Ca^++^ influx as well as neutrophil metabolism since both the inhibition of SOCE and of P2Y2-mediated ATP uptake diminished NET formation. Host cell invasion assays indicated that PMN-derived NETosis hampered tachyzoites from active host cell invasion, thereby inhibiting further intracellular replication. NET formation represents an early and effective mechanism of response of the innate immune system, which might reduce initial infection rates during the acute phase of cattle neosporosis.

## Introduction

*Neospora caninum* is an apicomplexan obligate intracellular parasite with comparable characteristics in structure and development to *Toxoplasma gondii* (1). It has a wide intermediate host range and is responsible for reproductive disorders mainly in cattle but is also associated with clinical reproductive and neural infections in dogs, horses, goats, sheep, and deer (2–4). In general, infections of apicomplexan parasites, such as *N. caninum*, underlie a complex adaptive immunological regulation (5–8); however, little is known on early host innate immune reactions occurring during primary *N. caninum* infection, despite the fact that early innate host defense reactions should be critical for the actual outcome of infection (7–13). In particular, polymorphonuclear neutrophils (PMN) play a key role in this respect since they are the most abundant innate immune cells in the blood and the first ones to be recruited to the site of infection (14–16). PMN own several effector mechanisms to combat and kill pathogens, such as phagocytosis, production of oxygen-based radicals known as reactive oxygen species (ROS), the excretion of antimicrobial peptides/proteins, and the synthesis of neutrophil extracellular traps (NETs) (17).

NETs are generally released *via* a novel PMN cell death process known as NETosis (17, 18). NETosis is known as a NADPH oxidase (NOX)-dependent mechanism (10, 12, 13, 17, 19), which leads to the extrusion of nuclear and cytoplasmic granule enzymes leading to the formation of DNA-rich networks adorned with different histones (H1, H2A/H2B, H3, H4) and antimicrobial granular effector molecules, such as neutrophil elastase (NE), myeloperoxidase (MPO), pentraxin, lactoferrin, cathepsins, gelatinase, bacterial permeability-increasing protein, peptidoglycan recognition proteins, calprotectin, and other leukocyte proteins (10, 16, 17, 20, 21). Classical NET formation [for review of pathways, see Ref. (17, 22, 23)] was initially proven to be signaled *via* the Raf–MEK–ERK-dependent pathways (24). In contrast to NOX-dependent NETosis, the recently described NOX-independent NETosis is associated with substantial reduced levels of ERK1/2 activation and weak Akt activation, whereas the activation of p38 MAPK is similar in both pathways (25). Irrespective of NOX-dependency, invasive pathogens may either be immobilized within NET-derived sticky DNA fibers or be killed *via* the locally high concentration of antimicrobial histones, peptides, and proteases (14, 21, 26). Moreover, Yipp et al. (27) recently demonstrated that PMN, which undergo NETosis without cell lysis, remain viable and retain their ability to phagocytise bacteria. In agreement with these findings, PMN also seem to be able to release small-sized NETs of mitochondrial origin without suffering cell death (28). By now, NETosis has been described to be triggered by different protozoan parasites *in vitro* and *in vivo*, such as *Plasmodium falciparum* (29), *Leishmania* spp. (30, 31), *Eimeria bovis* (12, 32), *Eimeria arloingi* (33), *T. gondii* (34, 35), *Besnoitia besnoiti* (11), *Cryptosporidium parvum* (13), *Trypanosoma cruzii* (36), and *Entamoeba histolytica* (37). In addition, monocyte-derived extracellular traps have recently been reported in response to tachyzoites of *B. besnoiti* (11) and *T. gondii in vitro* (35). Recent analyses on *Eimeria* spp. and *B. besnoiti*-induced NETosis confirmed their dependency on NOX, NE, MPO, CD11b, ERK1/2, p38 MAPK, and SOCE (12, 13, 32, 33). Moreover, blood vessel analyses of *P. falciparum*-infected patients (29) and intestinal tissue samples of *Eimeria*-infected goats and cattle also proved apicomplexan parasite-triggered NETosis to happen *in vivo* (38).

In contrast to ruminant eimeriosis, nothing is known on NET-based host innate immune reactions against *N. caninum*, although PMN and other leukocytes, such as macrophages and NK cells, seem to play a crucial role in neosporosis *in vivo* (9, 39–41). Thus, the aim of the present study was to analyze the capacity of *N. caninum* tachyzoites to trigger NETs and to unravel effector molecules and pathways being involved in this novel cell death process.

## Materials and Methods

### Ethics Statement

This survey was carried out in accordance to the Justus Liebig University Animal Care Committee guidelines. Protocols were approved by the Ethic Commission for Experimental Animal Studies of the Federal State of Hesse (Regierungspräsidium Giessen) (A9/2012; JLU-No. 521_AZ), in accordance to the prevalent European Animal Welfare Legislation: ART13TFEU and the current applicable German Animal Protection Laws.

### Parasites

All NET-related experiments were performed with tachyzoite stages of the apicomplexan parasite *N. caninum* [strain Nc1 (42)], which was cultivated *in vitro* as described elsewhere (7, 11). In brief, *N. caninum* tachyzoites were maintained by serial passages either in primary bovine umbilical vein endothelial cells (BUVEC) or permanent African green monkey kidney epithelial cells (MARC-145). Viable *N. caninum*-tachyzoites were collected from infected host cell layer supernatants, pelleted (400 × g, 12 min), washed thrice in sterile PBS, counted in a Neubauer hemocytometer (Marienfeld-Superior, Germany) and re-suspended in sterile RPMI 1640 medium (Gibco) until further experimental use.

### Host Cell Cultures

MARC-145 cell layers were maintained in cell culture medium DMEM (Sigma-Aldrich) supplemented with 1% penicillin (500 U/ml; Sigma-Aldrich), streptomycin (500 mg/ml; Sigma-Aldrich), and 10% FCS (Gibco) and cultivated at 37°C and 5% CO_2_ atmosphere until confluency. Confluent MARC-145 layers were infected with viable *N. caninum* tachyzoites (20 × 10^6^ parasites/25 cm^2^).

Isolation of primary BUVEC was performed according to the method reported by Taubert et al. (7). In brief, the umbilical cords retrieved from newborn calves were enriched with 1% penicillin–streptomycin (Sigma-Aldrich, St. Louis, MO, USA) and refrigerated in 0.9% HBSS–HEPES buffer (pH 7.4; Gibco, USA). Endothelial cells were isolated using 0.025% collagenase type II (Worthington Biochemical Corporation, USA), filling the lumen of the ligated umbilical vein and incubating for 20 min at 37°C in 5% CO_2_ atmosphere. Then, the umbilical vein was mildly massaged; the collagenase-cell suspension was retrieved and 1 ml FCS (Gibco, USA) was aggregated to inactivate the collagenase type II. After two centrifugations (400 × *g*, 10 min, 4°C), the isolated BUVEC were kept in complete ECGM (endothelial cell growth medium; PromoCell, Heidelberg, Germany), plated in 25 and 75 cm^2^ plastic culture flasks (Nunc, Roskilde, Denmark), and incubated at 37°C in 5% CO_2_ atmosphere until confluency.

### Isolation of Bovine PMN

Healthy adult dairy cows (*n* = 3) were bled by puncture of the jugular vein and 30 ml blood was collected in 50 ml sterile plastic tubes (Greiner), containing 0.1 ml heparin (Sigma-Aldrich) as anticoagulant. Approximately 20 ml heparinized blood was re-suspended in 20 ml PBS with 0.02% EDTA (Sigma-Aldrich), slowly layered on the top of 12 ml Biocoll Separating Solution^®^ (Biochrom AG), and centrifuged (800 × *g*, 45 min). After the extraction of plasma and mononuclear cells, the pellet was washed in 25 ml distilled water and gently shaken during 40 s to lyse erythrocytes. Osmolarity was rapidly normalized using an appropriate volume of Hanks balanced salt solution (4 ml, HBSS 10×, Biochrom AG). To complete the erythrocyte lyses, this step was repeated twice and the PMN were later re-suspended in RPMI medium (Gibco). Calculation and viability of the cells were performed in a Neubauer hemocytometer as described elsewhere (12). Finally, bovine PMN were cultured at 37°C and 5% CO_2_ atmosphere for 30 min until further use. As neutrophils have a short lifespan, PMN isolation was performed not exceeding 3 h after blood collection.

### Quantification of NETs

Bovine PMN (*n* = 3) were re-suspended in medium RPMI 1640 lacking phenol red and without serum and then confronted in duplicates with vital *N. caninum* tachyzoites (37°C, 4:1 ratio: 1 × 10^6^ *N. caninum* tachyzoites versus 2.5 × 10^5^ bovine PMN/200 μl). For NET blockage, the following inhibitors were used: the NOX-inhibitor DPI [10 µM, Sigma-Aldrich, according to Farley et al. (43)], the leukocyte elastase-inhibitor Suc-Ala-Ala-Pro-Val chloromethyl ketone [CMK; 1 mM, Sigma-Aldrich, according to Scapinello et al. (44)], the MPO-inhibitor 4-aminobenzoic acid hydrazide [ABAH; 100 µM, Merck, according Parker et al. (45)], the SOCE-inhibitor aminoethoxydiphenyl borate [2-APB; 100 µM, Sigma-Aldrich, according to Conejeros et al. (46)], UO126 as inhibitor of ERK1/2 [50 µM; Sigma-Aldrich, according to Muñoz-Caro et al. (12)], SB 202190 as specific inhibitor of p38 MAPK [10 µM; Sigma-Aldrich, according to Muñoz-Caro et al. (12)], the G-protein-coupled receptor (GPCR) antagonist NF-449 for P2Y2 blockage (GPCR-NF-449, 10 µM; Santa Cruz Biotechnology), and N-α-benzoyl-N5-(2-chloro-1-iminoethyl)-l-Orn amide for PAD4 inhibition (Cl-amidine, 200 µM, Merck). For blocking experiments, PMN were pre-exposed with the corresponding inhibitor in serum-free medium RPMI 1640 without phenol red (RT, GPCR-NF-449, and Cl-amidine: 120 min, all other inhibitors: 30 min) prior to exposure to *N. caninum* tachyzoites. To disrupt NETs and facilitate their DNA quantification, 50 µl of micrococcal nuclease buffer (New England Biolabs) including 0.1 U/μl micrococcal nuclease (New England Biolabs) was supplied to each well and incubated (15 min, 37°C). Next, all the samples were centrifuged (300 × *g*, 5 min). The supernatant of each sample was deposited in duplicate into a 96-well flat-bottom plate (100 µl per well). DNA from NETs was assessed using Pico Green^®^ (Invitrogen), an extracellular DNA-linking fluorescent stain. Fifty microliters of Pico Green^®^ (diluted 1:2,000 in 10 nM Tris buffer with 1 mM EDTA) was added to each well. NET production was quantified according to the fluorescence intensities obtained in the spectrofluorometric analysis (484 nm excitation wavelength and 520 nm emission wavelength) performed by an automated plate monochrome reader (Varioskan Flash^®^, Thermo Scientific). For negative controls, PMN in normal serum-free medium RPMI 1640 without phenol red were employed. Zymosan (1 mg/ml; Sigma-Aldrich) stimulated PMN served as positive controls according to Muñoz-Caro et al. (12). Diverse PMN-tachyzoites ratios were applied (1:1, 1:2, 1:3, 1:4) for dose-dependency evaluation. For analyses on the role of CD11b in parasite-triggered NETosis, the CD11b receptor was blocked *via* preincubation of bovine PMN in monoclonal mouse anti-bovine CD11b antibodies (MCA1425, diluted 1:5 in PBS; AbDSerotec, 30 min, RT). As antibody control, an irrelevant monoclonal antibody (mouse anti-bovine CD4, AbDSerotec) was used as described elsewhere (12). To resolve NET formation, DNase I (90 U/well, Roche Diagnostics) was supplemented 15 min before the end of incubation period.

### Visualization of NETs and Detection of Histones (H3), NE, MPO, and Pentraxin in *N. caninum* Tachyzoite-Induced NETs

Following PMN: *N. caninum* tachyzoite co-cultivation (ratio 1:4, 120 min, on 15 mm round glass coverslips pretreated with poly-l-lysine), fixation of the samples (4% paraformaldehyde, Merck, 15 min, 37°C), and three washings in PBS, the samples were blocked with BSA (2%, Sigma-Aldrich), incubated in antibody solutions (1 h, RT), and mounted on Prolong Gold^®^ with 4′-6-diamidino-2-phenylindole (DAPI) staining (Invitrogen, 1:1,000, 5 min, RT, in the dark). For the identification of antimicrobial peptides within extracellular DNA structures, the following antibodies were applied: anti-histone (H3) monoclonal [DyLight, ab139848, Abcam (1:1,000)], anti-MPO (Alexa Fluor 488, ABIN906866, https://Antibodies-online.com, 1:1,000), anti-NE (AB68672, Abcam, 1:1,000), and anti-pentraxin (SAB2104614-50UG, Sigma-Aldrich, 1:1,000) antibodies. The immunofluorescence images were taken by a digital camera from an inverted Olympus IX81 fluorescence microscope.

### NET-Related Host Cell Infection Experiments

To analyze the repercussions of parasite-induced NETs on tachyzoite infectivity, three different parallel experimental conditions were chosen: (1) *N. caninum* were cocultured with PMN (1:4 ratio, 2 h, 37°C) allowing for effective NET formation. (2) For infection control, an equal number of non-exposed tachyzoites was incubated in plain medium (2 h, 37°C). (3) The same amount of parasites were incubated with PMN (1:4 ratio, 2 h, 37°C) permitting a competent NET formation and furthermore treated with DNase I (90 U/well, addition of DNase I 15 min before the end of the incubation period) to resolve NET structures and to indirectly measure potential adverse effects of NETs on tachyzoite viability. In a next step, the tachyzoites of setups 1–3 were transferred to confluent BUVEC monolayers for infection (4 h, 37°C, 5% CO_2_ atmosphere). Overall, three different BUVEC isolates were used in this host cell invasion experiment. After incubation, BUVEC layers were washed to remove PMN and free tachyzoites. The infection rates were estimated microscopically (24 h p. i.) in 10 randomly selected vision power fields (400× magnification).

### Scanning Electron Microscopy (SEM)

Cattle PMNs were cocultured with viable tachyzoites of *N. caninum* (ratio: 4:1) for 10, 30, 60, and 120 min on 10 mm glass coverslips (Nunc) prepared with poly-l-lysine (Sigma-Aldrich). Then, cells were fixed in 2.5% glutaraldehyde (Merck), post-fixed in 1% osmium tetroxide (Merck), washed in distilled water, dehydrated, dried by CO_2_ treatment, and sputtered with gold as described elsewhere (11, 12). SEM samples were analyzed using a Philips XL30 scanning electron microscope (Institute of Anatomy and Cell Biology, Justus Liebig University Giessen, Germany).

### Statistical Analysis

Statistical analyses were performed by using Graph Pad Prism^®^ 6 software. One- or two-factorial analyses of variance (ANOVA) with repeated measures were applied to compare co-culture/stimulation conditions using a normal distribution of data. Dunnett’s multiple comparison tests were performed in dose and kinetic assays as follow-up test to ANOVA. For comparing enzyme activities, Tukey’s multiple comparison tests were used. Differences were considered as significant at a level of *p ≤ 0.05; **p ≤ 0.01; ***p ≤ 0.001, and ****p ≤ 0.0001.

## Results

### *N. caninum* Tachyzoites Trigger NET Formation

**Figure 1:**
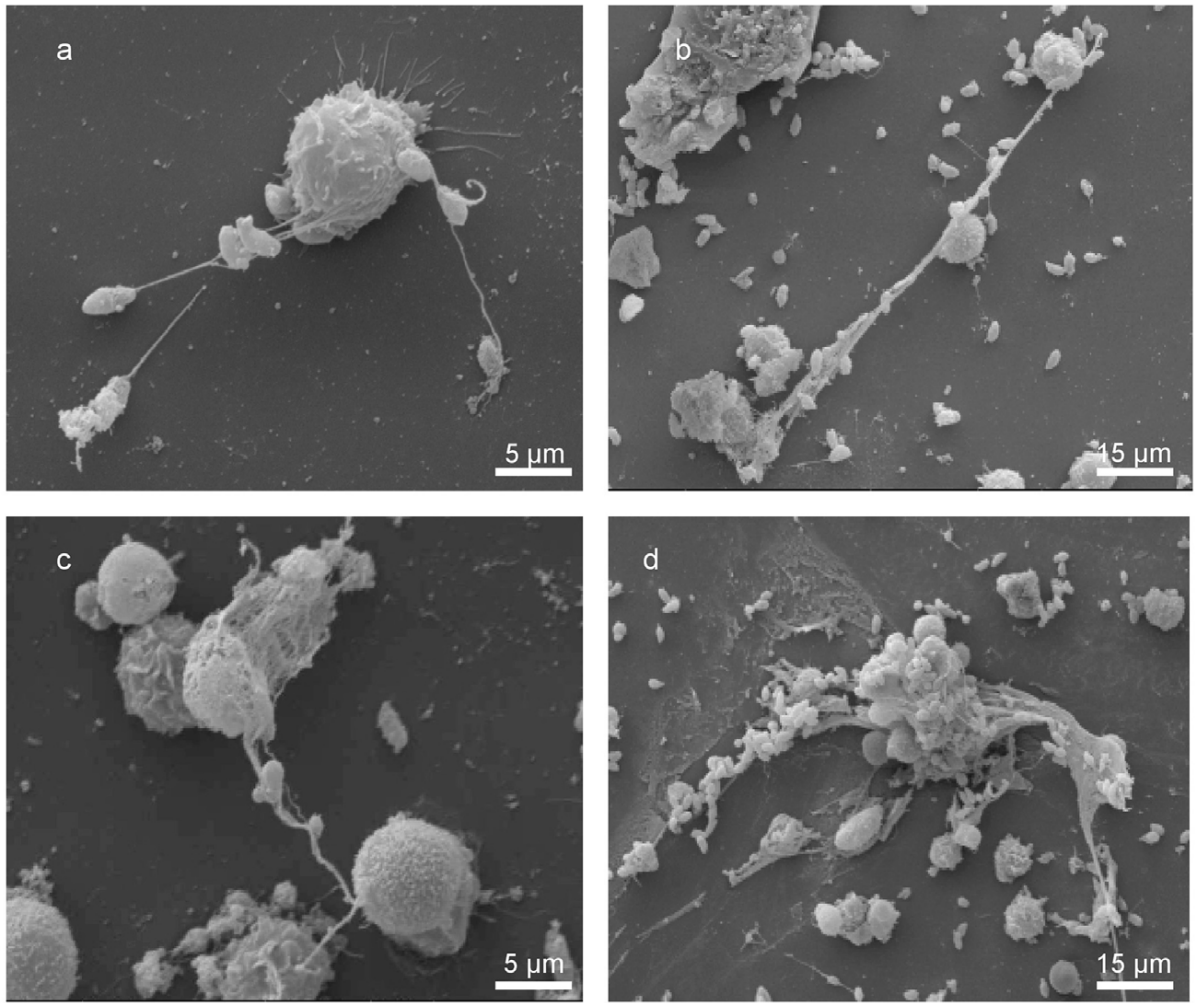
**Bovine neutrophil extracellular trap (NET) formation in response to *Neospora caninum* tachyzoite exposure**. Scanning electron microscopy analyses revealed NETs being formed by bovine polymorphonuclear neutrophils (PMN) after coculture with *N. caninum* tachyzoites for different time spans [**(A–C)** 30 min, **(D)** 60 min]. **(A–C)** Delicate PMN-derived filaroid structures being attached to tachyzoites and **(D)** conglomerates of several tachyzoites and a rather chunky meshwork of PMN-released filaments.

Scanning electron microscopy analyses revealed tachyzoite-triggered generation of a fine network of grosser and slimmer strands of fibers produced by bovine PMN and being solidly adhered to tachyzoites (see Figure 1). Kinetic studies reported several stages of NETosis: posterior to 30 min of exposure, smooth PMN-derived filament structures capturing tachyzoites were observed (Figure 1A). Here, PMN still presented undamaged cell morphology. Thereafter, parasites were entrapped in an extracellular network of long drawn-out fibers originating from disrupted PMN (Figures 1B,C) and conglomerates of *N. caninum* tachyzoites and rather thick and chunky meshworks of PMN-derived filaments (Figure 1D, 60 min) were observed.

**Figure 2:**
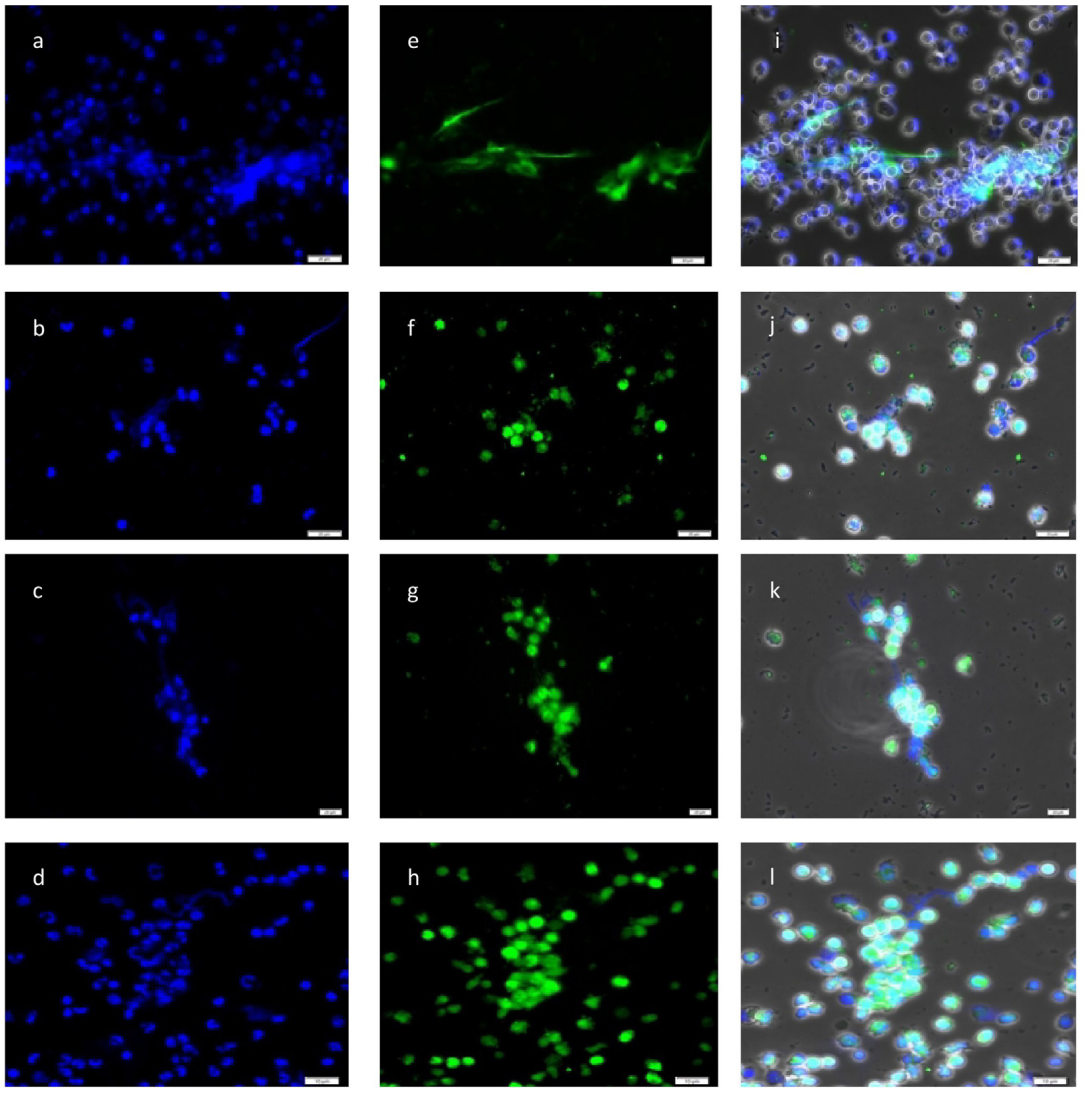
**Co-localization of DNA with histones (H3), neutrophil elastase (NE), myeloperoxidase (MPO), and pentraxin in *Neospora caninum* tachyzoite-induced neutrophil extracellular trap structures**. Cocultures of bovine polymorphonuclear neutrophil (PMN) and *N. caninum* tachyzoites (ratio 1:4, 120 min) were fixed, permeabilized, stained for DNA using Prolong Gold^®^ DAPI [blue, **(A–D)**], and probed for histones [green, **(E)**], NE [green, **(F)**], MPO [green, **(G)**], and pentraxin [green, **(H)**] using anti-histone (H3), anti-NE, anti-MPO, and anti-pentraxin antibodies and adequate conjugate systems. Areas of respective co-localization (merges) are illustrated in **(I–L)**.

DAPI-based fluorescence analyses further proved the presence of NET-like structures containing DNA (see Figure 2). Furthermore, *N. caninum* tachyzoites were located in close proximity to NETs and presumably were trapped in these extracellular chromatin-rich structures (Figure 2). Moreover, co-localization of extracellular chromatin with histones (H3), NE, MPO, and pentraxin in parasite-capturing structures validated the typical nature of NETs (Figure 2).

### *N. caninum*-Induced NETosis at Different Time-and Dose-Periods

**Figure 3:**
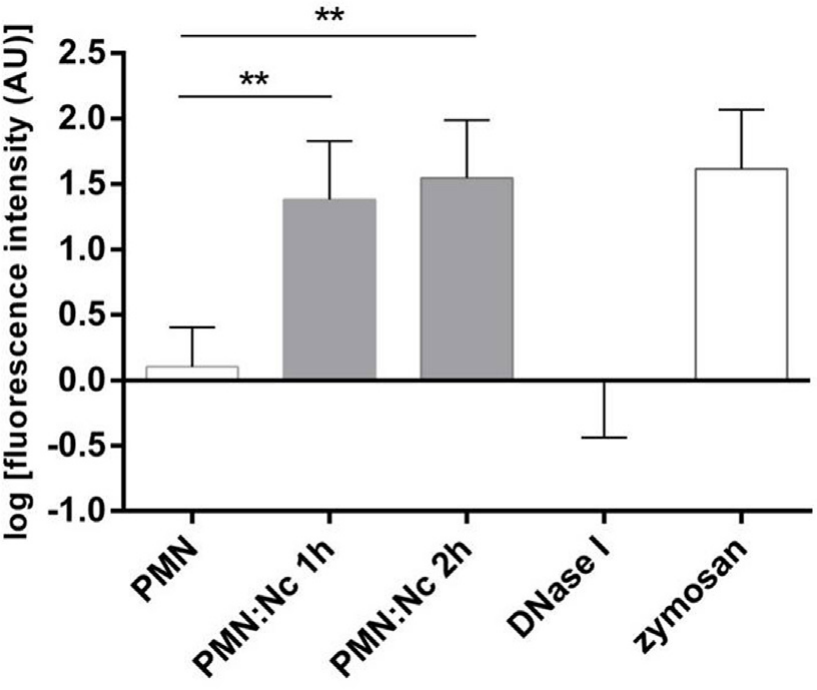
**Kinetics of *N. caninum* tachyzoites-triggered neutrophil extracellular trap (NET) formation**. Bovine polymorphonuclear neutrophils (PMNs) were incubated with tachyzoites (ratio 4:1), zymosan (1 mg/ml, positive control), or plain medium (negative control) for 1 and 2 h. To prove the DNA nature of NETs, the samples were treated with DNase I (15 min). After incubation, all samples were analyzed for extracellular DNA by quantifying Pico Green^®^-derived fluorescence intensities. Each condition was performed in duplicates. Arithmetic means of three PMN donors, minimum, and maximum. Differences were regarded as significant at a level of **p ≤ 0.01.

**Figure 4:**
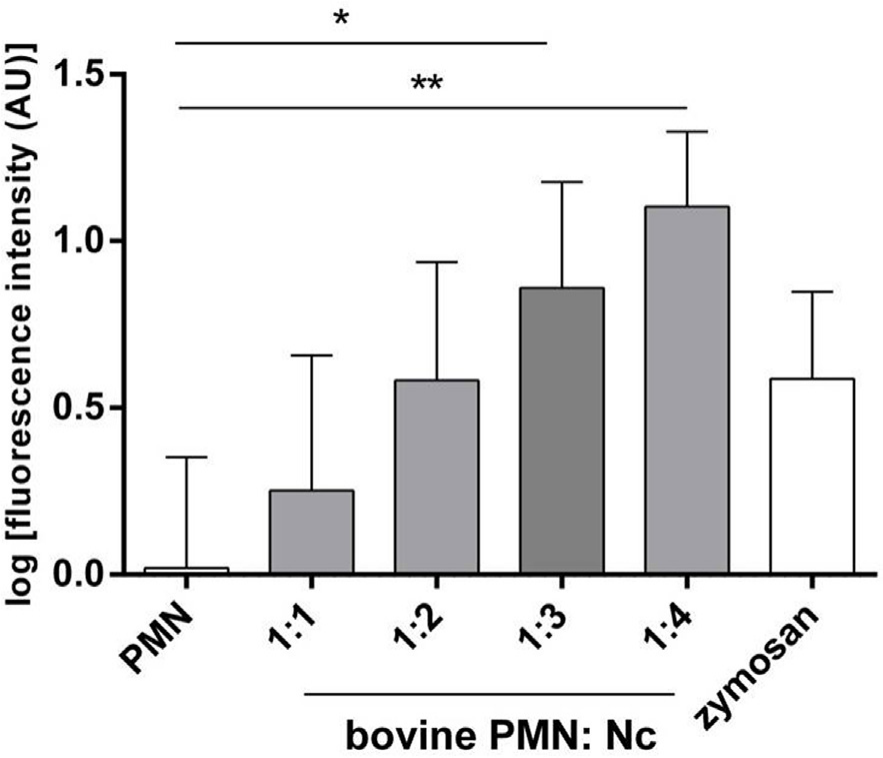
**Dose assays of *N. caninum* tachyzoites-induced neutrophil extracellular trap formation**. Bovine polymorphonuclear neutrophil (PMN) and *N. caninum* tachyzoites were incubated at different ratios (PMN: tachyzoites = 1:1, 1:2, 1:3, 1:4). After incubation (120 min), samples were analyzed for extracellular DNA by quantifying Pico Green^®^-derived fluorescence intensities. Each condition was performed in duplicates. Arithmetic means of three PMN donors, minimum, and maximum. Differences were regarded as significant at a level of *p ≤ 0.05 and **p ≤ 0.01.

**Figure 5:**
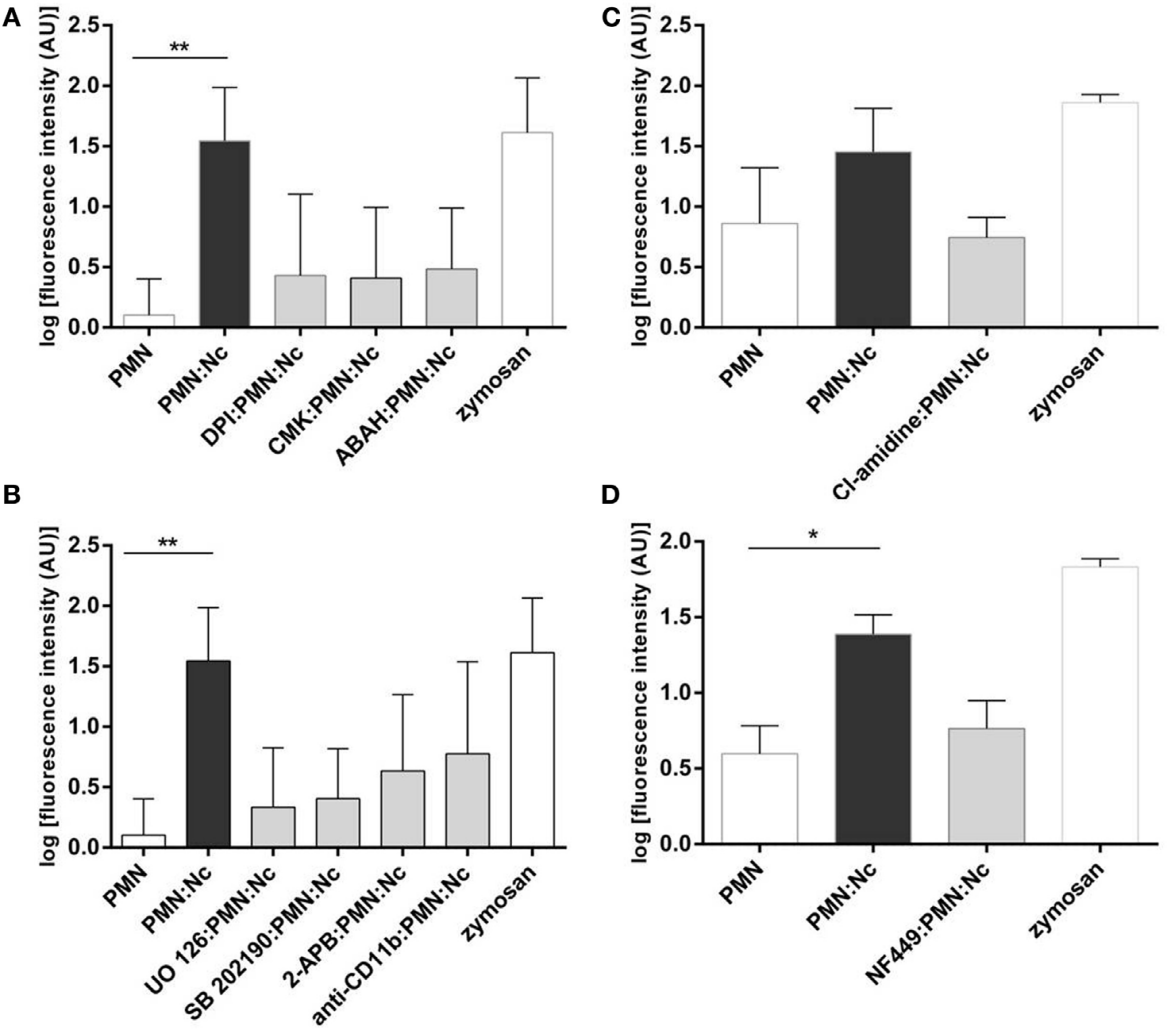
**Functional neutrophil extracellular trap (NET) inhibition assays**. Bovine polymorphonuclear neutrophils (PMN) were treated with the inhibitors of: **(A)** NADPH oxidase (DPI, 20 µM), neutrophil elastase (CMK, 1 mM), myeloperoxidase (ABAH, 100 µM), **(B)** ERK1/2 (UO126, 50 µM), p38 MAPK (SB 203580, 10 µM), SOCE (2-APB, 100 µM), CD11 monoclonal antibodies (diluted 1:5 in PBS), **(C)** PAD4 (Cl-amidine, 200 µM), and **(D)** P2Y2 (NF-449, 120 µM) prior to the exposure to tachyzoites (1:2 ratio). Non-treated PMNs were processed in parallel. Following *N. caninum* tachyzoites exposure, NET formation was determined by quantifying Pico Green^®^-derived fluorescence intensities (484 nm excitation/520 nm emission wavelengths). Stimulation of PMN with zymosan (1 mg/ml) was used for positive controls, plain medium served as negative control. Each condition was performed in triplicates for each PMN donor (*n* = 3). Differences were regarded as significant at a level of *p ≤ 0.05 and **p ≤ 0.01.

**Figure 6:**
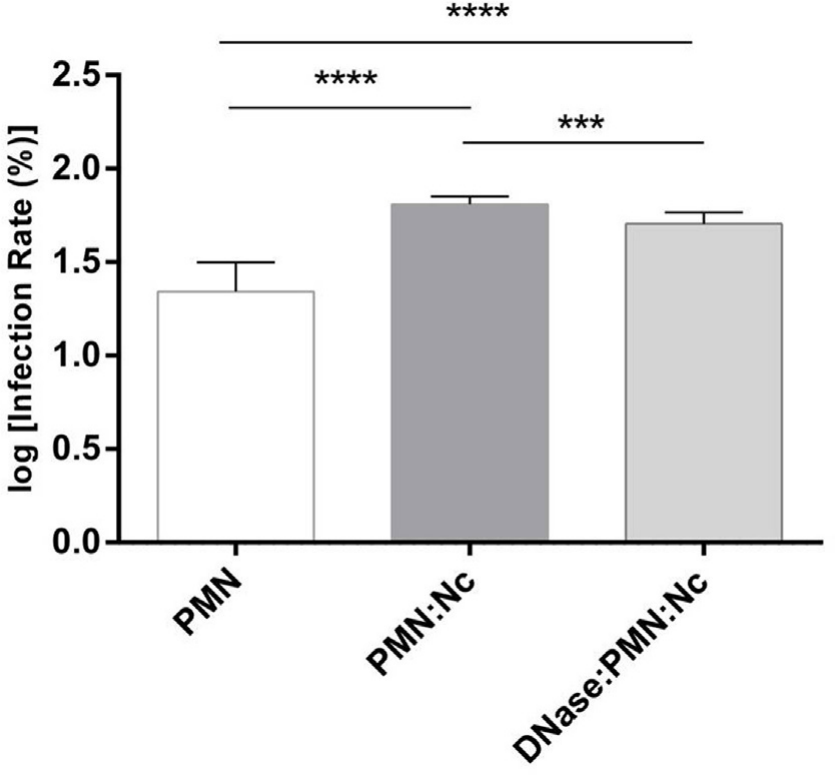
**Invasion assays of *N. caninum* tachyzoites after polymorphonuclear neutrophil (PMN) exposure**. *Neospora caninum* tachyzoites were cocultured with bovine PMN for 3 h (=PMN + Nc) allowing for effective neutrophil extracellular trap (NET) formation. In parallel, DNase I was supplemented to the tachyzoite-PMN-suspension 15 min before the end of the incubation period (=PMN + Nc + DNase I) to dissolve potential NET structures. The same number of non-treated tachyzoites in plain medium served as infection controls (=Nc only). Following incubation, the differentially treated tachyzoites were transferred to confluent bovine umbilical vein endothelial cell (BUVEC) monolayers for infection. Twenty-four hours p. i., the BUVEC layers were thoroughly washed and infection rates were estimated. Arithmetic means and SDs of three PMN donors and three BUVEC isolates, minimum, and maximum were calculated. Differences were regarded as significant at a level of ***p ≤ 0.001 and ****p ≤ 0.0001.

Neutrophil extracellular trap quantification experiments revealed *N. caninum* tachyzoites as strong triggers of NETosis, since these stages induced even stronger reactions than zymosan stimulation of PMN (= positive control, Figures 3–6). Kinetics on NETosis indicated a significant induction of NETs formation in both incubation time periods (1 and 2 h) compared with the negative control (*p* ≤ 0.01, Figure 3). As expected, DNase I treatments leading to NET disintegration reduced NETosis under the basal levels of the negative controls (Figure 3). Furthermore, increasing amounts of *N. caninum* tachyzoites led to enhanced levels of NET formation as significant differences were observed at a ratio of 1:3 and 1:4 (PMN: tachyzoites) in comparison with the negative controls (*p* ≤ 0.05 and *p* ≤ 0.01, respectively, Figure 4).

### *N. caninum*-Induced NETosis Is Reduced in Presence NOX-, NE-, and MPO Inhibitors

To further corroborate the molecular characteristics of *N. caninum*-mediated NETosis, functional blocking experiments with DPI, a potent blocker of NOX, were performed. The PMN treatment with DPI resulted in a clear reduction of tachyzoite-triggered NET formation (Figure 5A), despite that this diminution was statistically not significant. Additionally, treatments of PMN with MPO and NE inhibitors (ABAH and CMK, respectively) also generate a decrease of tachyzoite-triggered NET formation (Figure 5A) underlining the pivotal role of NE and MPO in this process.

### ERK1/2-, p38 MAPK-, SOCE-Signaling Cascades, and Bovine CD11b Are Involved in *N. caninum*-Induced NETosis

We here investigated the actual role of NET-associated molecular signaling pathways, receptors, and Ca^++^ influx in *N. caninum*-triggered NETosis. The use of inhibitors affecting ERK1/2-(UO126) and p-38 MAPK-(SB202190) signaling routes in functional NET-derived studies caused a diminishment of tachyzoite-induced NET production (Figure 5B), proving a key role of ERK1/2- and p38 MAPK in *N. caninum*-triggered activation of NETosis-related signaling pathways.

Given that NOX-dependent ROS synthesis in bovine neutrophils is being reported as a Ca^++^-associated process (12, 47, 48), we here furthermore tested whether *N. caninum*-triggered NETosis was influenced by SOCE. Treatments of PMN with 2-ABP produced a decrement of parasite-mediated NETs liberation (Figure 5B) proving that intracellular Ca^++^ mobilization is necessary for efficient parasite-induced NETosis.

Until now, no information is available on PMN receptors related to *N. caninum*-triggered NETosis. Therefore, we analyzed whether antibody-mediated blocking of bovine CD11b leads to the diminishment of tachyzoite-triggered NETosis. Indeed, pretreatment of PMN with anti-CD11b led to a decrease of NET formation, but, however, these reactions were barely no significant.

### Inhibition of PAD4 Signaling Pathway Diminished Strongly *N. caninum*-Triggered NETosis

Since no data are available on the role of histone hypercitrullination in parasite-induced NETosis so far, we here also intended to analyze whether the PMN exposure to Cl-amidine (200 µM), a specific inhibitor of PAD4, might have an impact on PAD4-derived histone hypercitrullination and chromatin decondensation during parasite-triggered NETosis. Cl-amidine pretreatment of bovine PMN resulted in diminished NET production when compared with non-treated but *N. caninum* tachyzoites-exposed PMN (Figure 5C). The same experiment was performed with zymosan as positive control (1 mg/ml) and negative controls (PMN cultured in plain medium alone).

### Inhibition of the ATP-Specific G-Protein Receptor P2Y2 Reduces *N. caninum*-Induced NET Formation

We further intended to determine whether *N. caninum*-triggered NETosis is an energy and ATP-dependent process as seen for other PMN effector mechanisms (49–51). Therefore, the blocker of the ATP-specific G-protein receptor P2Y2 (NF-449) was used here for functional inhibition experiments. In fact, PMN-pretreatment with NF-449 led to a reduction on parasite-triggered NETosis (Figure 5D) when compared to non-treated parasite-exposed PMN.

### *N. caninum*-Induced NET Formation Prevents Tachyzoites from Host Cells Infection

Host cell penetration is a vital requisite of the parasite *N. caninum* to survive and reproduce successfully within a host. Therefore, to analyze the effects of NET-mediated parasite entrapment on subsequent tachyzoite host cell infectivity, PMN-pre-exposed tachyzoites were transferred to BUVEC monolayers as suitable specific host cells and infection rates were later calculated. In the same way, an equal amount of tachyzoites, which had not been in contact with PMN before were used to infect BUVEC. The prior confrontation of parasites with PMN and subsequent NET development significantly (*p* ≤ 0.0001) prevented *N. caninum* tachyzoites from host cell invasion (Figure 6). As such, infection rates decreased from log 60% = 1.778 (resulting from non-exposed tachyzoites = infection controls) to log 20% = 1.3 induced by PMN-pre-exposed tachyzoites. To prove that this impairment was due to NETosis, parallel samples containing the same numbers of tachyzoites and PMN were treated with DNase I treatment (leading to NET disentangle) 165 min after PMN-tachyzoite-exposure (i.e., after a time period, which allowed efficient NET formation) and then used for BUVEC infections. As depicted in Figure 6, the infectivity of PMN-pre-exposed *N. caninum* tachyzoite was completely restored by DNase I treatment proving that, first, the ensnarement of tachyzoites within NETs hampered a large proportion of tachyzoites from active host cell invasion, and, second, that NETs had no lethal effects on tachyzoites of *N. caninum* within a period of 3 h.

## Discussion

Several protozoan parasites have been identified as potent NET inducers as well (11, 12, 29, 30, 32–35). To the best of our knowledge, we here describe, for the first time, the release of bovine NETs in response to the apicomplexan parasite *N. caninum*, which is known as an important abortive agent affecting not only beef and dairy cattle but also small ruminants worldwide (52–57).

In agreement with observations on other apicomplexan-triggered NETosis (11–13, 29, 32–35, 38, 58), we here report on NETs being attached to tachyzoites of *N. caninum*. The DNA-labeling of *N. caninum*-stimulated NETs confirmed the presence of chromatin structures of these extracellular networks. Moreover, the resolution of these mesh by DNase I treatments corroborated the DNA basis of *N. caninum*-mediated NETosis. NET-associated molecules, such as histones and antimicrobial peptides were detected in *N. caninum*-triggered NETs. Consistent to other reports on apicomplexan-induced NETosis (11–13, 33–35, 38, 58), co-localization assays demonstrated the concomitant existence of H3, NE, pentraxin, and MPO in *N. caninum*-caused NETs confirming molecular characteristics of NETs. Consequently, the key action of MPO and NE in *N. caninum*-achieved NETosis was proven through functional inhibition experiments, leading to a reduction of tachyzoite-mediated NETosis in both cases. Furthermore, we here delivered the first report on pentraxin involvement in apicomplexan-triggered NET formation. Pentraxin is a pivotal antimicrobial component of the mammalian host innate immune response, stored in PMN granules and, in common with MPO and proteinase 3, expressed on the apoptotic neutrophil surface while fighting against pathogens (59). During NETosis, pentraxin may participate in microbial recognition, thereby facilitating the trapping of pathogens. Interestingly, proteomic analyses revealed that pentraxin forms a complex with other NET components in human PMN, appearing as a binding molecule that enhances the actions of the different typical NETs molecules (60).

We here demonstrate that NOX participated in *N. caninum* tachyzoite-exposed bovine PMN since DPI treatments resulted in a decrease of parasite-driven NET formation. Similar findings have been reported from *E. bovis*- (12, 32), *T. gondii*- (35), *B. besnoiti*- (11), and *C. parvum*-triggered NETosis (13), emphasizing the importance of NOX-influence in parasite-mediated NETosis (61).

In contrast to *E. bovis*- (32) and *C. parvum*-related NETosis data (13), but according to *B. besnoiti*-induced NETs (11), neither a time- nor a dose-dependency of *N. caninum*-triggered NETosis was demonstrated as significant values were obtained only when each period of incubation (1 and 2 h) and the last two highest infection ratios (1:3 and 1:4) were compared with the negative controls. Furthermore, NET structures were demonstrated being firmly attached to tachyzoites of *N. caninum*, thereby supporting the quantitative data of tachyzoite entrapment showing that parasites were immobilized by extruded NETs. Consequently, *in vitro* host cell invasion experiments involving PMN-pre-exposed *N. caninum* tachyzoites unveiled a significant diminishment of their infectivity (40% reduction) for endothelial host cell. The crucial role of NETosis in this process was proven by the fact that the reduced infectivity could be restored by DNase I treatments. Moreover, this result proved that the tachyzoites were indeed not killed by extruded NETs as also demonstrated for several bacteria (62), protozoan parasites (11–13, 32, 33, 35), as well as metazoan parasites (13, 63, 64).

Taken together, these data confirm the capacity of NETs to hamper *N. caninum* tachyzoites from active host cell invasion *in vitro* by immobilizing them. Taking into account that tachyzoites of *N. caninum* obligatory must infect endothelial host cells *in vivo*, it seems reasonable to speculate that NETosis might represent an efficient defense mechanism during acute cattle neosporosis.

Considering that PMN-derived NOX-activation and subsequent ROS production is known to be Ca^++^/SOCE-dependent (65), we here employed the SOCE inhibitor 2-APB in NET-related functional studies, as described elsewhere (13, 47, 48). *N. caninum*-triggered NET formation proved to be influenced by SOCE since 2-APB applications limited the tachyzoite-induced NET formation. A Ca^++^ dependency on NET extrusion was also recently published for *E. bovis*- (12) and *C. parvum*-mediated NETosis (13) and for NETs release by human neutrophils in response to other non-parasitic stimulators (66).

The pivotal role of the Raf-MEK-ERK signaling pathways in the process of NETosis was first proven by Hakkim et al. (24). Here, functional inhibition experiments confirmed the importance of ERK1/2- and p38 MAPK-signaling pathways also for *N. caninum*-triggered NET formation. Thus, functional interference of these routes produced a reduction of tachyzoite-mediated NETosis (13). Corresponding findings on ERK1/2 and p38 MAPK have recently been reported on *T. gondii*- (34), *E. bovis*- (12), and *C. parvum*-induced NETosis (13), evidencing a general role of these signaling pathways in apicomplexan-derived NETosis.

Antibody-mediated blockage of CD11b failed to significantly reduce NETosis in the current study, thereby denying an essential role of CD11b in *N. caninum*-triggered NET formation.

PAD4, an enzyme that participates in the citrullination of histones, is known as an essential enzyme of NETosis (67, 68). PAD4-mediated hypercitrullination stimulates decondensation and deployment of chromatin, which allows adequate extrusion of NETs (67, 68). Consistently, functional inhibition experiments using Cl-amidine confirmed the role of PAD4-mediated histone hypercitrunillation for *N. caninum*-triggered NETosis as it resulted in a barely no-significant diminishment of tachyzoite-mediated NETosis.

The stimulation of purinergic receptors (e.g., P2X, P1, P2Y) generally promotes or inhibits cell responses through different signaling events in all kinds of mammalian cells and tissue inflammation (49, 50). Therefore, it requires the local release of extracellular ATP *via* pannexin 1 (PANX1) channels and/or the autocrine feedback regulation of this mechanism involving GPCR, such as P2Y2 (51, 69). These processes may result in the amplification of chemotactic signals through the binding to ATP and triggering PMN polarization/activation (69). In the current work, we, therefore, assayed for the role of P2Y2 in *N. caninum*-induced NETosis. Functional blockage of P2Y2 *via* NF-449 resulted in a decrease of tachyzoite-mediated NETosis, demonstrating for the first time the importance of this energy metabolism-related receptor in parasite-triggered NET formation. Given that P2Y2 also regulates PMN adhesion onto endothelial cells through the binding of ATP and UTP (70, 71) and since NETs were recently found adhered to *B. besnoiti*-infected endothelium (58), further investigation on the interrelationship of P2Y2 and *N. caninum*-induced NETosis will be of interest.

Overall, the current data demonstrated for the first time *N. caninum* tachyzoites as inducers of NET formation in cattle. Considering the life cycle of *N. caninum*, which includes endogenous parasite stages, such as tachyzoites and bradyzoites, exhibiting an obligatory intracellular replication, extracellular immobilization *via* NETosis might have implications in host cell invasion and, therefore, affecting the outcome of acute cattle neosporosis as previously postulated for closely related apicomplexan protozoa (10–12, 33).

## Conclusion

We identified *N. caninum* tachyzoites as NET inducers in bovine species, involving several molecular mechanisms. These data suggest that NETosis could be an important mechanism during the early host innate immune response against *N. caninum* in cattle.

## Availability of Data and Material

All data generated or analyzed during this study are included in this published article.

## Ethics Statement

This survey was carried out in accordance to the Justus Liebig University Animal Care Committee guidelines. Protocols were approved by the Ethic Commission for Experimental Animal Studies of the Federal State of Hesse (Regierungspräsidium Giessen) (A9/2012; JLU-No. 521_AZ), in accordance to the prevalent European Animal Welfare Legislation: ART13TFEU and the current applicable German Animal Protection Laws.

## Author Contributions

RV-B, LS, TM-C, and ZY performed the NET quantification and inhibition experiments. UG contributed in the performance of scanning electronic microscopy analysis. AT, JL, XZ, and CH cooperated in research design, data analysis, and manuscript’s review. All the authors checked and accepted the final manuscript.

## Conflict of Interest Statement

The authors ratified that they have no competing interests in the present study.
